# Review of statistical methods for survival analysis using genomic data

**DOI:** 10.5808/GI.2019.17.4.e41

**Published:** 2019-12-20

**Authors:** Seungyeoun Lee, Heeju Lim

**Affiliations:** 1 Department of Mathematics and Statistics, Sejong University, Seoul 05006, Korea; 2 Department of Statistics, University of Connecticut, Storrs, CT 06269, USA

**Keywords:** censoring, Cox model, Kaplan-Meier curve, machine learning, regularization, survival time

## Abstract

Survival analysis mainly deals with the time to event, including death, onset of disease, and bankruptcy. The common characteristic of survival analysis is that it contains “censored” data, in which the time to event cannot be completely observed, but instead represents the lower bound of the time to event. Only the occurrence of either time to event or censoring time is observed. Many traditional statistical methods have been effectively used for analyzing survival data with censored observations. However, with the development of high-throughput technologies for producing “omics” data, more advanced statistical methods, such as regularization, should be required to construct the predictive survival model with high-dimensional genomic data. Furthermore, machine learning approaches have been adapted for survival analysis, to fit nonlinear and complex interaction effects between predictors, and achieve more accurate prediction of individual survival probability. Presently, since most clinicians and medical researchers can easily assess statistical programs for analyzing survival data, a review article is helpful for understanding statistical methods used in survival analysis. We review traditional survival methods and regularization methods, with various penalty functions, for the analysis of high-dimensional genomics, and describe machine learning techniques that have been adapted to survival analysis.

## Introduction

Survival analysis arises in many applied fields such as medicine, biology, engineering, epidemiology and economics. Survival time is defined as a time to event of interest such as death, relapse of disease, unemployment and completion of a task. The main characteristic of survival time is that it is censored due to the end of study or withdrawal during the period of study because we cannot follow-up the exact survival time for those who are still alive at the end of the study, or who are lost to follow-up during the period of study. However, it is known that the survival time of censored individuals is at least longer than the censoring time. There are many different types of censoring such as right-, left-, and interval-censoring. The most popular censoring mechanisms are right censoring, in which the lower limit of the exact survival time is observed, while the upper limit is observed for left censoring, and both lower and upper limits are used for interval-censoring. More details about censoring mechanism are described [1,2].

Many statistical methods have been developed for estimating survival functions, comparing survival curves between two groups, and modeling the survival data by regression, for association with risk factors, such as demographic and clinical predictors. In survival analysis, nonparametric statistical inference is more extensively used to estimate the survival function, and compare survival curves between two or more groups. For example, both the Kaplan-Meier (KM) estimator [3], for a survivor function, and a log-rank test [4], for comparison of survivor functions, are derived by a nonparametric approach. However, if the appropriate distribution for survival data is assumed or pre-specified, the parametric approach is more appropriate. When the association of survival time with various risk factors is the main interest, the most popular model is a Cox regression [5], based on a semiparametric approach, since the effect of predictors on the hazard rate is parametrically specified, while the baseline hazard function is unspecified. A variety of parametric approaches are also available under the assumed survival distributions, such as an accelerated failure time (AFT) model. Overall, all survival analysis approaches should take into account a censoring mechanism, when a statistical inference is made [1,2].

In the early 21st century, DNA microarrays for characterizing gene expression patterns have been used to more distinctly classify diseases, including cancer subtypes [6,7]. For example, diffuse large B-cell lymphoma (DLBCL) is regarded as a clinically heterogeneous disease, in which 40% of patients respond well to current therapy, having prolonged survival, with the remaining 60% being mostly unresponsive, with low survival rates [7]. It was further found that DLBCL survival rates differ significantly between germinal center B-like DLBCL and activated B-cell DLBCL. As a result, genome-scale views of gene expression provide a new approach for identifying and classifying cancers more clearly, by comparing gene-specific survival curves. Since the amount of gene expression data is extraordinarily large, relative to sample sizes, significance difference testing of gene expression levels between normal and cancer patients, for a single gene, yields multiple testing problems. Early on, Bonferroni correction was applied to address the multiple testing problem in most cases, but only a few, among thousands, of genes were detected, due to extremely conservative Bonferroni correction [8]. Alternatively, the false discovery rate [9] was proposed to adjust for multiple testing, using criteria that was less conservative than Bonferroni correction. Such large amounts of genomic data yielded the problem of “curse of dimensionality,” due to the dimensionality of microarrays being much larger than the sample sizes (p >> n), unlike traditional (“pre-genomic era”) cases (p << n). This translated the analysis of gene expression data into regression modeling, as related to a variable selection problem in the fields of statistics and bioinformatics. In the frame of the regression model, many penalized functions have been proposed to fit high-dimensional genomic data, such as lasso [10], ridge [11], and elastic-net [12].

The human genome was determined to possess a sequence of 3 billion nucleotides, as determined by Human Genome Project in 2003 [13]. With the development of high-throughput technologies, such as microarrays, single nucleotide polymorphism arrays, proteomics and RNA sequencing (RNA-seq), biological data collected from the same individual is referred to as ‘omics’ data. Information from omics data can be used as diagnostic markers, by physicians, to predict the health status of individual patients, and the accruement of such data will progressively increase in the future. Based on these considerations, it is very important to precisely identify significant disease biomarkers from such huge amounts of complex omics data. Machine learning (ML) techniques are widely used to model nonlinear and complicated gene-to-gene interactions, and improve predictability, in various practical domains. For survival analysis, ML methods have been adapted to effectively handle censored information, and accurately construct prediction models, using high-dimensional data.

Since many clinicians can easily assess statistical programs such as SAS, SPSS, and R for analyzing survival data, a review article is helpful for understanding statistical methods used in survival analysis. We will briefly review the basic concepts and theories in survival analysis by explaining a KM estimator for the survival function, a log-rank test for comparing two-sample survival curves, and a Cox model for studying association with risk factors. Then the most widely used regularization methods will be described for high-dimensional genomic data analysis. Finally, a comprehensive review of omics ML methods will be given, with a short conclusion.

## Traditional Survival Methods

### Basic functions

Three functions are used to characterize the distribution of survival time, namely, the survival function, hazard function, and probability density function [1,2]. Let *T* be a non-negative random variable representing the survival time, and let *f(t)* and *F(t)* be the probabilities of density function and cumulative distribution function of *T*, respectively. Then, the survival function, *S(t)*, the hazard function, *h(t)*, and the cumulative hazard function, *H(t)* are specified as:

$S(t)=1-F(t)=P(T>t)=\int_{t}^{\infty }f(s)ds\;h(t)=\underset{dt\to 0}{lim}\frac{P(t\leq T<t+dt|T\geq t)}{dt}\;H(t)=\int_{0}^{t}h(s)ds$

In addition, the following relationship is easily derived, and if any one of these functions is known, then the other functions can be uniquely determined.

$f(t)=\;-\frac{dS(t)}{dt},\;H(t)=-ln\;[S(t)],\;S(t)\;=\;exp(-H(t))=exp[-\int_{0}^{t}h(s)ds]$

In survival analysis, *h(t)* is more useful than *f(t)* in estimating *S(t)*, because it is the conditional probability of experiencing a specific event instantaneously, given that the survival remains up to that time. It also uses information from the censored observations by considering the conditional probability that they are survived up to that time, through censoring.

### KM estimator

For estimating the survival function, the KM [3] estimator has been most widely used in many clinical studies. The KM estimator is a nonparametric estimator, in the sense that no assumption for the survival distribution is needed, and uses the conditional probabilities at each distinct death time, given those subjects at risk just prior to that time, which include all the information about both death and censoring.

Let *T* and *C* be the survival and censoring times, respectively. Then, the observed time is defined as $\tilde{T}=min(T,C)$, and an indicator for uncensored observation is *δ=I(T ≤ C)*. The survival data are represented as $\left({\tilde{T}}_{i},\delta_{i}),\;i=1,\;\ldots \;,\;n.\;\mathrm{Let}\;0\;=\;t_{0}\;<\;t_{1}\;<\;t_{2}\;<\;\cdots \;<\;t_{d}(d\leq n\right)$ be the ordered distinct death times. Let *d_i_* be the number of deaths at time *t_i_* and let *Y_i_* be the number of subjects at risk at time *t_i_*-, which only counts the subjects still surviving to just prior to time *t_i_*. Suppose that *p_i_* denotes the conditional probability that a death occurs at time *t_i_*, given those still alive just prior to *t_i_*. Then the estimate of *p_i_* is given as ${\hat{p}}_{i}=\frac{d_{i}}{Y_{i}}$, and the estimator for the conditional survival probability is $1-{\hat{p}}_{i}=1-\frac{d_{i}}{Y_{i}}$. Using the conditional survival probability, the survival function at time *t_i_* can be represented as follows: $S(t_{i})=P(T>t_{i}│T>t_{i-1})P(T>t_{i-1}|T>t_{i-2})\cdots P(T>t_{1}|T>t_{0})P(T>0)=\prod_{j=1}^{i}(1-p_{j})$

From this, the KM estimator is given as $\hat{S}(t)=\prod_{t_{i}\leq t}(1-\frac{d_{i}}{Y_{i}})$, which is the sequential product of the reverse hazard function, which is easily estimated by $\frac{d_{i}}{Y_{i}}$, at each distinct death time. Unlike the complete data, the KM estimator is obtained as the product of the reverse hazard rates, by taking proper account of censoring into the risk set, *Y_i_*. Many results about the properties of the KM estimator have been studied, relating to its asymptotic distribution, self-consistency, and efficiency [14,15].

### Log-rank test

For clinical trials, it is common to assess the efficacy of a new drug or treatment compared to a placebo group. If the response variable is completely observed, either a t-test or Wilcoxon test is most suitable to solve this two-sample testing problem. However, neither of these is suitable for censored survival data. A log-rank test was originally proposed for one-sample problems [16], but was easily extended to the nonparametric two-sample comparisons of censored data [4,17]. The main idea of the log-rank test is to sum up the difference between the observed and expected number of deaths, across a time duration, and standardize it by its standard deviation. The expected number of deaths is calculated under the null hypothesis of equal survival function. The asymptotic distribution of the log-rank test is derived from the conditional distribution of the occurrence of a death, given that an individual survives just prior to each observed time. Under the null hypothesis of equal survival functions, this conditional distribution of the occurrence of a death is hypergeometric, and its expectation and variance are easily derived. To calculate the log-rank test, consider a 2 ×2 table at each distinct death time *t_i_* (Table 1 ).

Here, *d_i_* and *Y_i_* denote the number of deaths and individuals at risk at time *t_i_*, respectively, and * d_ji_* and *Y_ji_, (j*=1,2) denote the number of deaths and individuals at risk for the corresponding group, respectively. Then, the conditional distribution of *d*_1*i*_, given *Y_i_*, is hypergeometric, under the null hypothesis of equal survival functions. The log-rank test is then given as follows: ${\chi_{LR}}^{2}=\frac{{\left\{\sum_{i=1}^{d}\left(d_{1i}\;-\;Y_{1i}\;\frac{d_{i}}{Y_{i}}\right)\right\}}^{2}}{\sum_{i=1}^{d}\frac{Y_{1i}Y_{2i}}{Y_{i}-1}\frac{d_{i}}{Y_{i}}\left(1-\frac{d_{i}}{Y_{i}}\right)}$

where *d* is the total number of distinct deaths from the two groups. Assuming the independence of *d*_1*i*_ across times, it is known that the log-rank test has an asymptotic chi-square distribution, with one degree of freedom, under the null hypothesis. As shown in the equation above, the log-rank test is powerful when the two hazard rates are proportional across times, since it takes the sum of the differences between the observed and expected number of events. However, if the two hazard rates cross or are not proportional, the log-rank test yields lower power and other tests, such as Kolmogorov-Smirnov, Cramer-von Mises type tests, or median tests, are preferred [18-20].

### Cox regression model

Most statistical methods of survival analysis have focused on finding risk factors among many possible demographic, environmental, and clinical variables, and predicting the survival probability of the patient with a certain disease. For censored survival data, a Cox regression model [5] has been most widely used to study the association of risk factors with survival time, in much of clinical and biomedical research. It was proposed, on the basis of the hazard rates, as follows: h(tX)=h0(t)exp (Xβ)

Here, *X* represents the vector of risk predictors, and h(tX) is the hazard function with covariate *X*, while *h_0_(t)* is a baseline hazard function with *X*=0. By dividing both sides by the baseline hazard function and taking the logarithm, the Cox model can be rewritten as the following linear regression model: loghtXho(t)=Xβ.

Then, the regression coefficient, *β*, can be interpreted as the relative hazard rate between two individuals as one unit of *X* changes. For example, if *X*=1, for the treatment group, and *X*=0, for the placebo group, the hazard rate of those who take treatment is *β* times those in the placebo group. For *β*<0, the treatment is considered beneficial, whereas for *β*>0, it is considered deleterious.

For the estimation of *β*, the partial likelihood function of a Cox model was proposed [21], in which only *β* is involved in both score function and Fisher information, while the unspecified baseline hazard function is not considered. In other words, the statistical inference for *β* is made on the basis of the partial likelihood, regardless of the baseline hazard function. Only when one is interested in estimating the survival function from a Cox model, should we consider the estimation of the baseline hazard function, which is described by a Breslow’s estimator [16].

Since the log ratio of two hazard rates does not depend on time, as shown by the equation above, this derivation is known as a proportional hazards (PH) model. This proportionality between hazard functions is a strong assumption in real-life situations and requires evaluation by a goodness-of-fit test. However, since the Cox model is a fundamental basis for association studies in survival analysis, it has been further generalized to the stratified Cox model and the time-dependent Cox model, in which the proportionality assumption is not valid.

On the other hand, an AFT regression model is also widely used to fit the relationship between survival time and risk factors, in which the log survival time is specified as linear combinations of risk factors, with error random variable. According to the distribution of the error random variable, the parameters of the AFT model are estimated by maximum likelihood methods [1]. Compared to the parametric AFT model, the Cox model is considered semiparametric, as it consists of the baseline hazard function and *e^Xβ^*, in which no specific distribution is assumed for *h*_0_*(t)*. The effect of the regression coefficient of a Cox model is interpreted as the relative hazard rate of the corresponding risk factors, whereas the effect of the regression coefficient of an AFT model is interpreted as an accelerated factor of the survival time. Other types of regression models include Aalen’s additive model [22], and partly parametric, additive risk models [23].

## Regularization Methods for Analyzing Genomic Data

### Penalized Cox models

Since microarray data is used in association studies with survival time, a number of studies have been published regarding solutions to high-dimensional problems, in which there are too few observations for too many variables. To identify significant disease-associated genes, single-gene approaches were applied to circumvent the high-dimensional problem with adjustment of multiple testing problem. However, there remain limitations to the single-gene approach, because it is too simple to explain complex associations between genes, environments, and diseases.

For processing high-dimensional genomic data, one solution is to regularize selection of significant variables, via penalized models. Such regularization may rely on an assumption of sparsity, i.e., that only a few genes have significant effects on diseases, among thousands of genes [24]. With a Cox regression model, a variety of penalized models have been proposed, including lasso-Cox, ridge-Cox, and elastic-net Cox [10-12] to maximize the partial likelihood under the different penalty functions. The estimates $\hat{\beta }$ for the regression parameters, of the three models, are obtained by minimizing the negative partial log-likelihood function, subject to penalties, as follows: ${\hat{\beta }}_{Lasso}\;=\;argmin\left\{-\sum_{i=1}^{n}\delta_{i}(X_{i}\beta -log(\sum_{i=1}^{n}exp(X_{i}\beta )))+\lambda \sum_{k=1}^{p}\left|\beta_{k}\right|\right\}\;{\hat{\beta }}_{Ridge}\;=\;argmin\left\{-\sum_{i=1}^{n}\delta_{i}(X_{i}\beta -log(\sum_{i=1}^{n}exp(X_{i}\beta )))+\lambda \sum_{k=1}^{p}\left|{\beta_{k}}^{2}\right|\right\}\;{\hat{\beta }}_{EN}\;=\;argmin\left\{-\sum_{i=1}^{n}\delta_{i}(X_{i}\beta -log(\sum_{i=1}^{n}exp(X_{i}\beta )))+\lambda \sum_{k=1}^{p}\left|\beta_{k}\right|+(1-\alpha )\sum_{k=1}^{p}{\beta_{k}}^{2}\right\}$

Here *δ_i_* is an indicator for the uncensored observation, and *λ* is called a “tuning parameter” that controls the degree of regularization. When *λ*=0, there is no regularization, whereas when *λ*→∞, the coefficients tend to be more regularized. As shown above, the lasso imposes a *L*_1_- penalty on the regression coefficients, the ridge imposes a *L*_2_- penalty, and the elastic-net model combines the two penalties. In general, the lasso performs well in selecting significant genes, among many thousands, but tends to select only one gene from any specific group of genes, and does not care which one is selected when pairwise correlations, between genes, are very high. Furthermore, for the case of *p≫n*, the lasso selects at most *n* variables, due to the nature of the convex optimization problem. On the other hand, the ridge method, originally proposed to solve multicollinearity between predictors, is not appropriate for the variable selection problem. Thus, when the correlation between genes is more of interest rather than variable selection, the ridge penalty is more appropriate. The elastic-net method takes the weighted penalties of both lasso and ridge and performs better than the other two methods, in the sense that it selects more variables than *n*, even in the instance of *p≫n* cases, and considers correlations between genes. For example, it was shown in analysis of prostate cancer patient data that the elastic-net model had the smaller test error, with the same number of variables as the lasso [12]. Subsequently, other various modifications relating to regularization have been proposed, such as adaptive lasso-Cox [25], fused lasso [26]. and least angle regression elastic net [27].

## ML Methods for Analyzing Censored Survival Data

Recently, ML techniques have been rapidly adapted to a variety of fields, for automatically analyzing huge amounts of data. The basic concept of ML is to make the computer “learn” from repeated input data, and recognize hard-to-discern patterns from large, noisy, or complex data. This ML approach is well-suited to construct a predicted model when there are both nonlinear and complex interactions, among several features. Thus, ML has been widely applied to cancer prognosis and prediction, for medical applications. Predicted survival rates are particularly interesting, as they are part of a growing trend toward personalized medicine.

Although ML techniques have been developed and applied to artificial intelligence and data mining [28], these methods have also been translated into statistical ML and rapidly adapted to many disciplines related to statistical problems [29]. Subsequently, many good textbooks and website lectures for ML techniques have been disseminated [30,31], allowing many researchers to understand the fundamental theories and methods of statistical ML, as well as easily accessible (e.g., “open source”) programs.

Many types of ML systems exist, depending on whether they are trained with human supervision, such as supervised, unsupervised, semi-supervised, and reinforcement learning. Among those, the most interesting one is supervised learning, whose main task is classification, and predicting a target variable such as survival time. In this review, we will focus on the following ML techniques that are adapted to survival data: survival trees, support vector machines (SVMs), and ensemble methods such as bagging survival trees, random survival forests, Cox boosting, and artificial neural networks (ANNs).

### Survival trees

Decision trees have been useful for the classification and prediction of a wide range of applications, because it requires few statistical assumptions, readily handles various data structures, and provides easy and meaningful interpretation. Several studies on the practical and theoretical aspects of tree-based methods were developed, and the classification and regression tree (CART) software program has made tree-based methods popular, applied statistical tools [32]. Regression trees construct an optimal decision tree, by partitioning a set of predictors to accurately predict a dichotomous outcome. For example, clinicians are often interested in classifying small numbers of groups of patients with differing prognostics.

Survival trees were first proposed by adapting most of the CART paradigm for analyzing censored survival data by minimizing the within-node variabilities in survival time. Alternatively, the other approach for survival tree construction has been developed by maximizing the difference in survival between “child nodes,” as measured by two-sample test statistics, such as a log-rank [33,34].

The components of the survival tree algorithm consist of rules for growing the tree, pruning the tree, and choosing a tree of the appropriate size. The most common rule for growing and pruning a tree is a log-rank test, which tests for dissimilarity in survival between two groups. More properties for measures of splitting have been studied in detail [33,34]. Once the tree has been split recursively to pre-specifying nodes, the optimally pruned subtrees are found by using a measure of the tree’s performance, such as a split-complexity measure. Finally, the optimal tree size is selected by resampling or permutation procedures. The software for survival tree analysis is available at (https://CRAN.R-project.org/package=rpart).

Recently, survival trees have been constructed for analyzing multivariate survival time data, when the subjects under study are either naturally clustered or experience multiple events (namely, recurrent times) [35]. A multivariate survival tree constitutes a modified CART procedure, to model the correlated survival data by using a splitting statistic to handle the dependence between survival times. There are two main approaches for analyzing multivariate survival times; the marginal approach and the frailty model approach. The marginal approach uses a robust log-rank statistic, while the frailty model approach is based on either the semiparametric gamma [36] or parametric exponential frailty models [37], which lessen the computational burden. The multivariate survival tree can be implemented via (https://CRAN.R-project.org/package=MST).

### Support vector machines

SVMs are powerful ML methods, capable of performing linear or nonlinear classification, regression, and outlier detection. SVMs were first proposed for binary classification problems, and then subsequently extended to regression, clustering, and survival analysis. The main idea of SVMs is to maximize the margin between two classes and find a separating hyperplane that minimizes misclassification. The separating SVM hyperplane not only separates the two group classes, but also stays as far away from the close observations possible. The observations located on the edge of the separating hyperplane are known as the support vectors that fully determine the classification.

Although linear SVM classifiers are efficient and perform well in many cases, high-dimensional datasets are often not separated by a linear SVM classifier. To handle both nonlinear and high-dimensional datasets, the SVM classifier uses a high-dimensional kernel function to make the original dataset linearly separable. SVMs were subsequently extended to regression and censored survival data. By considering the penalty for censored observations, the SVM method for regression of censored data (namely, SVCR) was proposed [38], and shown to have superior performance. When SVCR is compared to the classical parametric models, for several survival analysis datasets, it has lower value of the average absolute errors, and has a computational run time that is favorable to other methods.

The support vector regression for censored data (SVRc) was subsequently proposed to take into account an asymmetric penalty (or loss function) for censored and non-censored data. In terms of the concordance index and the hazard ratio, SVRc performed better than the Cox PH model in five real-life survival analysis datasets [39]. The software for survival SVMs is available at https://CRAN.R-project.org/web/packages/survivalsvm/.

### Ensemble methods

Ensemble methods are based on the wisdom of “the crowd,” i.e., a new classifier produced by aggregating or voting from a group of classifiers. For example, a group of decision tree classifiers can be produced from different random subsets of a training set. To make a prediction, we may obtain predictions of all the individual decision tree classifiers, and then predict the class with the most votes. Multiple classifiers often predict better than individual classifiers, and appropriately weigh several classifiers, to improve predictability. In this section, we briefly review three ensemble methods: bagging survival trees, random survival forests, and Cox boosting.

### Bagging survival trees

The terminology of bagging stands for “bootstrap aggregating,” and the random sampling from a training set is performed repeatedly, with replacement known as B bootstrap samples. We then obtain a set of survival trees, based on B bootstrap samples, and define a new predictor by aggregating all predictors from a set of survival trees. In general, the new predictor can be a statistical mode for classification or an average for regression problem. It is known that the ensemble predictor reduces both bias and variance, compared to a single predictor.

For censored survival data, the averaged point predictor, such as the mean or median survival time, is of minor interest, compared to the predicted conditional survival probability of a new observation. Based on the bagging survival trees, one single KM curve is calculated from the observations identified by the “leaves” of *B* bootstrap survival trees [40]. Although the predicted survival probabilities aggregated from multiple survival trees are not easily interpreted, they are based on similar observations, classified by repetition of learning samples, in the aggregated set. We also note that the bagging survival trees depend on both the number of bootstrap samples and the size of multiple trees. As usually shown in ensemble methods, bagging survival trees results in a conditional survival probability prediction that is better than a single survival tree, in terms of the mean integrated squared errors, even when the censoring proportion is 50%. The software for using bagging survival trees is available at (https://CRAN.R-project.org/web/packages/ipred/).

### Random survival forests

Like bagging survival trees, the random survival forest is based on random bootstrap samples from a training set, but also allows extra randomness when growing trees. Instead of searching for the same set of variables when splitting a node, random survival forests search for the best variables among a random subset of variables, and these variables are used to split the node by maximizing the log-rank statistic. Similar to the classification and regression problems, random survival forests are an ensemble learner formed by averaging a number of base learners. In survival settings, the base-learner is a survival tree, and the ensemble is a cumulative hazard function formed by averaging individual tree’s Nelson-Aalen’s cumulative hazard function [41].

When implementing random survival forests, a primary interest is how to select a random subset of variables as candidates for splitting a node. The traditional variable selection in random forests is based on the variable importance (VIMP), a measurement of the increase (or decrease) in prediction error, for the forest ensemble, when a variable is randomly ‘noised up’. However, VIMP is based on the prediction error, and varies considerably, depending on the data with a high-dimensional problem. Alternatively, as described in Ishwaran et al.’s study [41], the minimal depth is introduced as a new high-dimensional variable selection measure, which assesses the degree of prediction of a variable by its depth, relative to the root node of a tree. In a single decision tree, important variables are likely to appear closer to a root of the tree, while unimportant variables are often closer to leaves. Thus, the variable’s importance can be estimated by the average depth, at which it appears across all trees in the forest. A smaller value of the minimal depth corresponds to a more predictive variable, and the effective way of using the minimal depth is well demonstrated as a high-dimensional survival problem. The software for using random survival forests is at https://CRAN.R-project.org/web/packages/randomSurvivalForest/.

### Cox boosting

Boosting was originally based on combining multiple weak learners into one strong learner, as proposed in the ML community, especially for classification problems [42]. As a useful ensemble, boosting has been successfully translated into the field of statistics [43], and extended to statistical problems such as regression and survival analysis.

The main idea of boosting is to update the predictors sequentially, which at each iteration, fit a weak predictor of the previous version of the data, as updated by minimizing a pre-specified loss function. The obtained value provides a small contribution used to update a new predictor, and all contributions result in a final predictor. Unlike bagging and random survival forests, boosting is a sequential learning technique, implying that it cannot be parallelized. Historically, the AdaBoost method was first proposed [42], which sequentially adds new predictors to an ensemble, by boosting the misclassified cases and reweighting all the cases, at every iteration. This addition stops when the desired number of predictors is reached, or when a perfect predictor is found. On the other hand, Gradient Boosting creates a new predictor based on the residual errors made by the previous predictor, and the small amount of updating is added sequentially, to improve prediction. For coping with the problem of analyzing high-dimensional data, component-wise boosting has also been adapted to survival analysis.

In survival analysis, most boosting methods have focused on the Cox model, by using gradient boosting, with a loss function derived from the Cox partial likelihood function, as used in the popular R-packages *mboost* and *CoxBoost* [44]. Both *mboost* and *CoxBoost* are based on gradient boosting, but differ in the sense that *mboost* is an adaptation of model-based boosting, whereas *CoxBoost* adapts likelihood-based boosting. The *mboost* algorithm computes the direction in which the slope of the partial log-likelihood is steeper, and then estimates an updated parameter, by minimizing the residual sum of squares of the multivariate regression model, with shrinking of the penalized parameter. This procedure is iteratively performed until the stopping criterion is met. On the other hand, the *CoxBoost* algorithm uses a negative *L*_2_-norm penalized partial log-likelihood, and updates the estimates of the parameter by maximizing this penalized partial log-likelihood, with a tuning penalty.

Furthermore, to improve the Cox model’s prediction, an offset-based boosting approach was adapted to allow for a flexible penalty structure, including unpenalized mandatory variables, when clinical covariates should be included with high-dimensional omics data [45]. Combining clinical and microarray information improves the predictive performance of the Cox model, compared to a microarray-only model.

There are two main parameters to be considered in a boosting procedure: the first controls the weakness of the estimators, known as a penalty or boosting step. The other parameter specifies how many boosting iterations should be performed, which is related to avoiding overfitting, and in component-wise boosting, controls the sparsity of the model. Beside these, other approaches to survival analysis include *L*_2_ boosting [46], using inverse probability of censoring weighting, and the boosted AFT model [47]. Recently, a new boosting method for nonparametric hazard estimation was proposed, when time-dependent covariates are present [48].

### Artificial neural networks

For specific detection and prediction of breast cancer risk, several ANN models have been developed over the last few decades [49,50]. Although the ANN method has been long applied to cancer prognosis and prediction, it has some drawbacks in that it cannot be intuitively interpreted, unlike the decision tree. However, with the development of computing techniques and the generation of large amounts of omics data, ANN is becoming more widely used, and can also be extended to deep neural networks.

In review of the literature, the ANN method was first applied to survival analysis [51], for modeling prostate cancer survival data with only four clinical predictors. Subsequently, many other researchers implemented ANN methods to predict patient survival, using high-dimensional microarray expression data [52,53].

Recently, a neural network extension of the Cox regression model, “Cox-nnet,” was proposed to predict patient prognosis from high-throughput transcriptomics data [54]. Cox-nnet is composed of one hidden layer, and the output layer is used to construct the Cox regression model, based on the activation levels of the hidden layer. In Cox-nnet, high-dimensional genomic data is optimized by dropout regularization, and the model is trained by minimizing the partial log-likelihood, using back-propagation. Furthermore, Cox-nnet reveals more information about relevant genes and pathways, by computing feature importance scores from the Cox regression model. The advantage of Cox-nnet is that it overcomes the weaknesses of the ANN model, which is regarded to be a “black box,” with a lack of interpretable relationships between the hidden layers and the outcome variable. The code for Cox-nnet is available at http://github.com/lanagamire/cox-nnet.

On the other hand, neural network techniques have been developed to overcome the PH assumption of the Cox model, to allow more general relationships between survival time and high-dimensional omics data. For example, the multi-task logistic regression model was developed [55], in which a series of logistic regression models were fitted on different time intervals to estimate the survival function, without any of the aforementioned assumptions. However, this multi-task logistic regression model only involved linear relationships. Consequently, a neural multi-task logistic regression model was proposed by involving a deep learning architecture to fit nonlinear dependencies [56]. For implementation of this model, the open-source libraries TensorFlow and Keras [31] may be used, with many state-of-the-art techniques of deep neural network methods, including initialization, optimization, activation functions, and miscellaneous operations such as batch normalization and dropout regularization. Overall, a deep neural network approach would be more useful to cover nonlinear and complex dependencies between survival time and high-dimensional omics data, without relying on Cox PH assumptions. However, many issues remain, regarding the choice of schemes and hyper-parameters, which still may yield many possible combinations for fitting the target output.

## Analysis of a Real Data Set

In order to illustrate three different types of methods reviewed, we applied these methods to a real dataset from The Cancer Genome Atlas (TCGA) Genome Data Commons (GDC) portal (https://portal.gdc.cancer.gov) [57]. This real dataset consists of 125 pancreatic ductal adenocarcinoma (PDAC) with the RNA-seq and clinical information. For the RNA-seq data, an Illumina HiSeq instrument (San Diego, CA, USA) was used for mRNA profiling. In the sample selection procedure, non-PDAC samples were removed and samples with a survival time less than 3 months were removed, since the cause of death may not be due to PDAC. As a result, we analyzed 124 PDAC patients, among which there were 61 female and 63 male patients. The median survival time was 598 days, and the censoring proportion was 41%. The average and standard deviation of age was 64.56 years and 10.91 years, respectively.

We applied the preprocessing procedure to RNA-seq data of 56,716 genes annotated. The relative log expression (RLE) normalization method was adopted to control the gene length bias. The RLE method was implemented in R package (v3.5) “DESeq2” (v1.22.2) [58]. After RLE normalization, the genes with zero proportion larger than 80% were filtered out [59] and the number of remaining genes was 37,406. In addition, we fitted a Cox model with a single clinical variable and selected only 11 variables among 40 variables, in which 10 variables have the significant p-value less than 0.1 and a variable of sex is included with p-value of 0.312 as shown in Table 2.

**Fig. 1.**
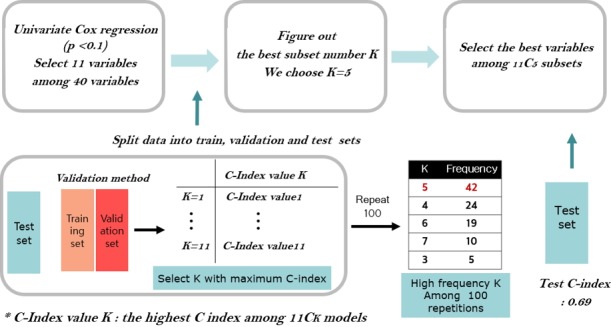
Clinical variable selection scheme.

First, we applied the traditional method by fitting a Cox model with eleven clinical variables, in which five clinical variables were selected by 3-fold cross validation as shown in Fig. 1. Secondly, we applied the penalization method by using lasso and elastic-net penalties with 37,406 RNA-seq data. For the lasso penalization, eight genes were selected whereas eleven genes were selected for the elastic-net penalization. Thirdly, we applied ML methods such as SVMs, random survival forest and Cox boosting with five clinical variables and the selected eight or eleven genes depending on either lasso or elastic-net, respectively. The C-index [60] is used to evaluate the prediction of each method and presented in Table 3.

As shown in Tables 3 and 4, the prediction models considering both clinical and gene variables have the larger C-index than those with either clinical or gene information. Especially when only gene information is considered, the value of C-index is lower than the model with only clinical variables. When comparing two penalties of lasso and elastic-net, the gene variables from the lasso penalty seem to be more informative on the prediction of the survival time than those from the elastic-net penalty. When comparing the four methods of Cox model, SVMs, random survival forest and Cox boosting, both the Cox model and Cox boosting have almost identical C-index values and perform better than SVMs and random survival forest. The SVMs seem to be sensitive to the choice of the penalty function whereas the random survival forest tends to be robust. From this result, the lasso penalty yields better prediction with less gene variables than the elastic-net penalty. However, it is not possible to find out the optimal penalty which always yields to the best prediction for all methods. Instead, many possible methods should be applied to a real data with several penalties to give the best prediction.

## Conclusion

In this article, we reviewed statistical methods for survival analysis, focusing on the adaption of traditional methods, regularizations, and ML. We introduced how a KM estimator can determine a survival function, how the log-rank test can compare two survival curves, and use of the Cox regression model. Though there are many other estimators, tests, and models available for survival analysis, the abovementioned methods are the most popularly applied, and the Cox model in particular, is widely adapted for regularization and ML techniques.

With development of high-throughput biological data acquisition, a variety of high-dimensional omics data has rapidly accumulated, allowing for more personalized information used for detection, and prediction of survival probability. Presently, many ML techniques have increasingly been combined with traditional survival methods, and regularization approaches, to provide more accurate diagnosis and prognostic decision-making, in clinical practice (i.e., “precision medicine”). We also briefly reviewed more useful and applicable ML techniques such as survival trees, SVMs, bagging, random survival forests, Cox boosting, and ANNs. Especially, ANNs are more powerful when the relationship between covariates and the outcomes are not linearly correlated and do not restrict any specific functional relationship between covariates and outcomes. By allowing more hidden layers and a variety of flexible open-source deep learning techniques, ANNs are increasingly used for detection and prediction, in biomedical fields.

As described in this article, the classical statistical methods in survival analysis have been well adapted to the ML techniques, to improve survival predictability. This trend will continue to be substantially expanded, in conjunction with deep learning techniques, which are now explosively utilized in many domains (including artificial intelligence).

## Figures and Tables

**Table 1.** 2 × 2 table at time *t_i_* for calculating the log-rank test statistic

Group	Dead	Alive	Risk set
Treatment	*d*_1*i*_	*Y*_1*i*_-*d*_1*i*_	*Y*_1*i*_
Placebo	*d*_2*i*_	*Y*_2*i*_-*d*_2*i*_	*Y*_2*i*_
Total	*d*_*i*_	*Y*_*i*_-*d*_*i*_	*Y*_*i*_

**Table 2.** Result of univariate Cox model for clinical variables

Clinical variable	p-value^a^		
Age at initial pathologic diagnosis	0.103		
Maximum tumor dimension	0.001		
Sex	0.312		
Anatomic neoplasm subdivision	0.017		
Surgery performed type	<0.001		
Residual tumor	0.108		
T stage	0.096		
N stage	0.053		
Radiation therapy	0.004		
Postoperative rx tx	<0.001		
Person neoplasm cancer status	<0.001		

a p-value from a univariate Cox model.

**Table 3.** Comparison of C-index of using clinical and lasso gene variables

	C-index		
Method	Clinical	Lasso genes	Clinical + Genes
Cox model	0.75 ± 0.06	0.60 ± 0.14	0.84 ± 0.03
SVM	0.65 ± 0.12	0.50 ± 0.03	0.74 ± 0.07
RSF	0.73 ± 0.11	0.56 ± 0.08	0.78 ± 0.08
Cox boosting	0.75 ± 0.06	0.60 ± 0.13	0.84 ± 0.03

Values are presented as mean ± standard deviation.

SVM, support vector machine; RSF, random survival forest.

**Table 4.** Comparison of C-index using clinical and elastic net gene variables

Method	C-index							
	Clinical	E-N gene	Clinical + Genes					
Cox model	0.75 ± 0.06	0.64 ± 0.09	0.79 ± 0.07					
SVM	0.65 ± 0.12	0.54 ± 0.14	0.64 ± 0.16					
RSF	0.73 ± 0.11	0.61 ± 0.07	0.77 ± 0.08					
Cox boosting	0.75 ± 0.06	0.64 ± 0.09	0.80 ± 0.05					

Values are presented as mean ± standard deviation.

SVM, support vector machine; RSF, random survival forest.
